# A bibliometric analysis of liver cancer bone metastases: advances in mechanisms of occurrence and treatment options

**DOI:** 10.3389/fonc.2025.1594848

**Published:** 2025-07-10

**Authors:** Jiyong Wei, Yanni Lan, Guipeng Lan, Ronghe Gu

**Affiliations:** ^1^ Department of Spine Surgery, The First People’s Hospital of Nanning, The Fifth Affiliated Hospital of Guangxi Medical University, Nanning, China; ^2^ Department of Pharmacy, The People’s Hospital of Guangxi Zhuang Autonomous Region and Guangxi Academy of Medical Sciences, Nanning, China; ^3^ Department of Bone Surgery, The Eight People’s Hospital of Nanning, Nanning, China

**Keywords:** bibliometric, liver cancer, bone metastasis, molecular, resection

## Abstract

**Background:**

Liver cancer ranks as the sixth most frequently diagnosed cancer and is the third leading cause of cancer-related deaths worldwide. Its poor prognosis is mainly due to tumor metastasis. Bone metastasis commonly occurs in the advanced stages of liver cancer and can significantly impact patients’ quality of life and prognosis. However, current treatment methods for bone metastasis resulting from liver cancer have significant limitations. This article aims to review the latest research and trends in global studies on bone metastasis associated with liver cancer.

**Methods:**

This study used the Web of Science Core Collection to gather 3,347 articles on liver cancer with bone metastasis published from 2004 to 2024.The study applied bibliometric analysis methods, including CiteSpace, VOSviewer, and other tools, to examine countries, institutions, journal authors, keywords, and references.

**Results:**

From 2004 to 2024, we collected 3,845 records, including 3,347 articles and 498 reviews from 92 countries. Publications had shown exponential growth, peaking in 2021 with an annual growth rate of 8.3%. The research involved 13,188 organizations. China had the most publications (n=970, 29%), followed by the United States (n=648, 19.4%), Japan (n=317, 9.5%), and Germany (n=228, 6.8%); Canada had a notably higher rate of international collaboration at 44.8%. In total, 830 journals contributed to this research. The most cited journal was “J Clinical Oncol” with 4,275 citations, while “New Engl J Med” had the highest impact factor at 158.5. The research involved 23,710 authors, with Wang, Xin and Zhang, Chao being the authors with the most publications. Wang Z published the first study on this topic in 2007. In 2008, Zhang Y, Wang Y, and Zhang J also began researching this area and have continued to be actively involved since then. The study referenced a total of 79,935 sources, with the paper by Sung H et al., published in CA Cancer J Clin in 2021, achieving the highest citation rate of 42.46.

**Conclusion:**

This study systematically summarizes research findings on liver cancer with bone metastasis from 2004 to 2024, It also identifies and predicts global research hotspots and trends. In the future, the molecular mechanisms of liver cancer with bone metastasis will be a major research topic. The expansion of surgical treatment options and the advancement of innovative therapies will benefit more patients.

## 1 Introduction

Liver cancer poses a significant threat to public health around the world. In 2022, liver cancer was the sixth most commonly diagnosed cancer worldwide and the third leading cause of cancer-related deaths. The International Agency for Research on Cancer (IARC) reported 865,269 new liver cancer cases and 757,948 related deaths globally in 2022. The incidence of liver cancer was slightly higher in males than in females. Additionally, the likelihood of developing the disease increases significantly for individuals over 50 years old, with notable regional differences (1–3). Recent advancements in clinical diagnosis and treatment have introduced a range of therapeutic options for liver cancer (4–10). Each treatment method has its unique advantages and limitations, and they often share similar indications. These advancements have significantly increased the overall survival time for patients with early-stage liver cancer. However, most patients are diagnosed at intermediate or advanced stages, missing the opportunity for surgical intervention. Even when surgical resection is attempted, patients face a high risk of postoperative recurrence, complicating their prognosis (11, 12).

Recently, the incidence of bone metastasis from liver cancer has increased significantly. It has been reported that bone metastasis has become the second most common site of metastasis from liver cancer, accounting for 25% of extrahepatic metastases (13). The reasons why the skeleton is the most common metastasis site in various cancers are still unclear. This may be due to the bone marrow microenvironment, which supports cancer cell growth. Studies indicate that the mechanisms behind bone metastasis from liver cancer may involve epithelial-mesenchymal transition, angiogenesis, the bone microenvironment, factors related to bone metastasis, osteoclast activation, interactions between tumor cells and bone marrow stromal cells, regulatory non-coding RNAs, and the immune microenvironment (14–17). Currently, screening, diagnosing, and treating bone metastasis from liver cancer are significant global challenges. This highlights the need for multidisciplinary collaboration, personalized treatment plans, and comprehensive management throughout the treatment process (18–21).

Bibliometric analysis is a quantitative research methodology that employs statistical and computational tools to evaluate scholarly literature, aiming to uncover research trends, collaborative networks, and knowledge structures within a specific field. Its significance lies in its ability to transform fragmented bibliographic data into interpretable patterns, enabling researchers to identify emerging hotspots, assess disciplinary development, and guide future investigative directions.

This study presents the first comprehensive bibliometric analysis of liver cancer with bone metastasis, assessing literature from 2004 to 2024. This analysis offer a blueprint of the current research framework on liver cancer with bone metastasis, especially for newcomers to the field. It includes an introduction to recent research focuses and trends and aims to help them quickly master the latest technologies and explore potential avenues for future research:

1. Utilizing leading analytical methods in the field of bibliometrics, this study explores the current research status and hotspots in the field of liver cancer with bone metastasis, and predicts future research trends.
2. Exploring the current research status of the main research directions in liver cancer with bone metastasis, including molecular mechanisms, monitoring targets/techniques, treatment methods, and prognosis.

## 2 Materials and methods

### 2.1 Database and search methodology

**Figure 1:**
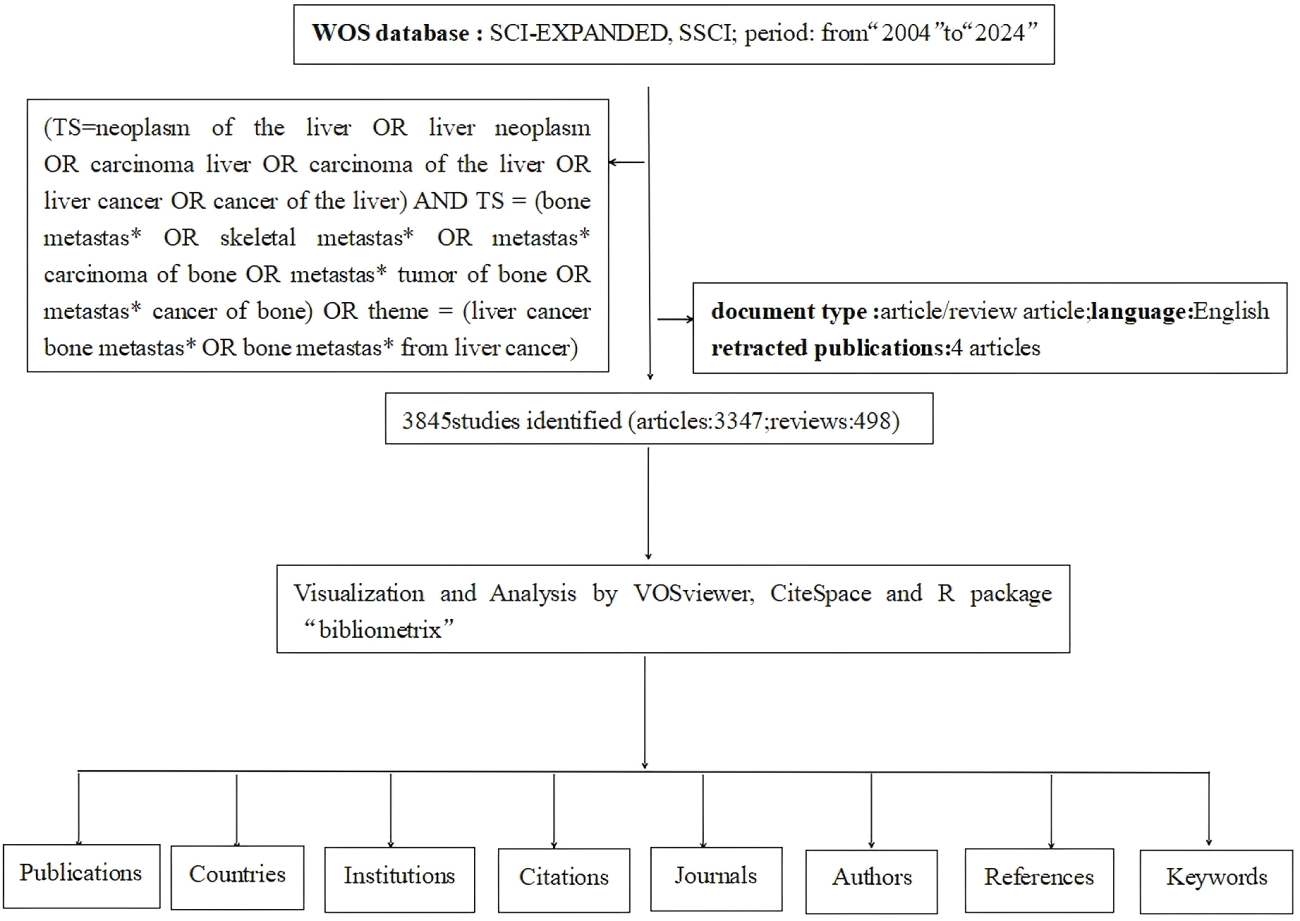
Detailed flowchart steps of the search strategy in screening publications.

This study utilized the Web of Science Core Collection (WOSCC) as the literature search database. Visual mapping was conducted using software such as CiteSpace, VOSviewer, Bibliometric tools, and Microsoft Excel. The search terms were “TS =(neoplasm of the liver OR liver neoplasm OR carcinoma liver OR carcinoma of the liver OR liver cancer OR cancer of the liver) AND TS = (bone metastas* OR skeletal metastas* OR metastas* carcinoma of bone OR metastas* tumor of bone OR metastas* cancer of bone) OR TS = (liver cancer bone metastas* OR bone metastas* from liver cancer)”. The following adjustments were made: Document type: “Article/Review Article”; Language: “English”; Time: “from 2004.01.01 to 2024.10.31”. The field label “TS = Topic” was used with the publication date as the selection criterion and duplicate documents removed, resulting in the final retrieved material (**Figure 1**).

### 2.2 Data analysis

Bibliometric analysis is a method used to describe and analyze the dynamics and progress of research in a particular field. With the assistance of modern computing technology, it visualizes the analysis results in the form of knowledge maps, which not only facilitates data analysis however also helps to uncover the intrinsic connections within this information.

Bibliographic coupling, co-authorship, co-occurrence, citation, and theme patterns of each publication were examined using VOSviewer (version 1.6.20) (22), including network visualization, overlay visualization, and density visualization, it is characterized by simple mapping and visually appealing images.

Professor Chaomei Chen developed CiteSpace (version 6.3 R1) (23), as a tool to analyze the evolutionary process of knowledge. A set theory-based method standardizes data to measure the similarity of knowledge units. This method is used within time slices to create regional views and timeline graphs. This approach clearly outlines the evolutionary process of knowledge and the historical span of literature within a cluster. As a result, it helps to understand the development and trends in the field.

We utilize R-bibliometrics software (Version R 4.0) to analyze research hotspots and trends, focusing on keywords, trending topics, and thematic maps (24).

## 3 Results

### 3.1 Analysis of the number of publications and citations

**Figure 2:**
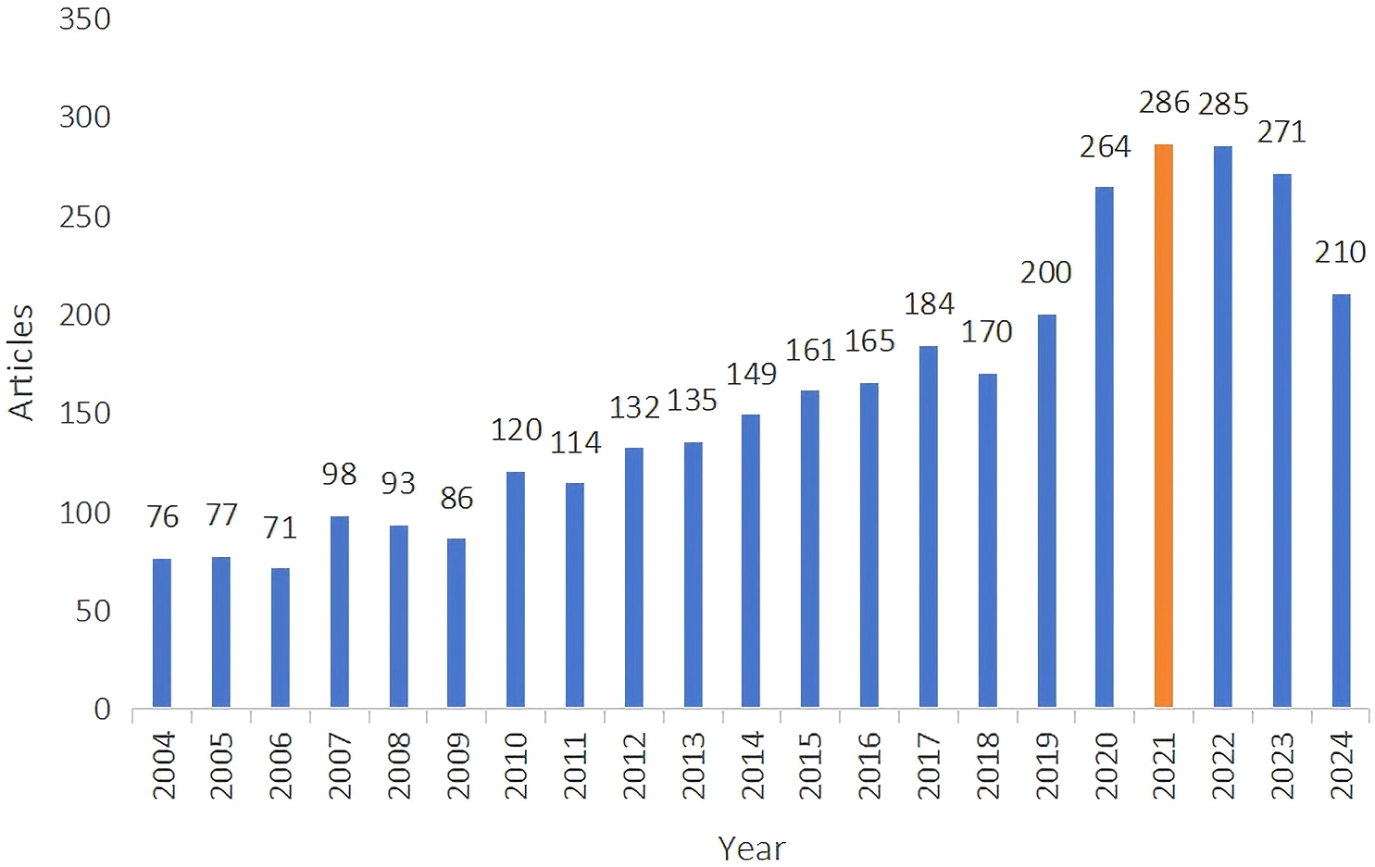
Annual trends of global publication outputs in liver cancer with bone metastasis research.

We retrieved a total of 4,068 papers related to liver cancer with bone metastasis. After screening, we selected 3,845 papers, which included 3,347 “articles” and 498 “reviews,” as illustrated in **Figure 2**. we noted that the number of publications increased each year from 2004, and reached a peak of 286 papers in 2021, with an annual growth rate of 8.3%, since then, the number of publications has remained relatively stable.

### 3.2 Analysis of the contributions of countries

**Figure 3:**
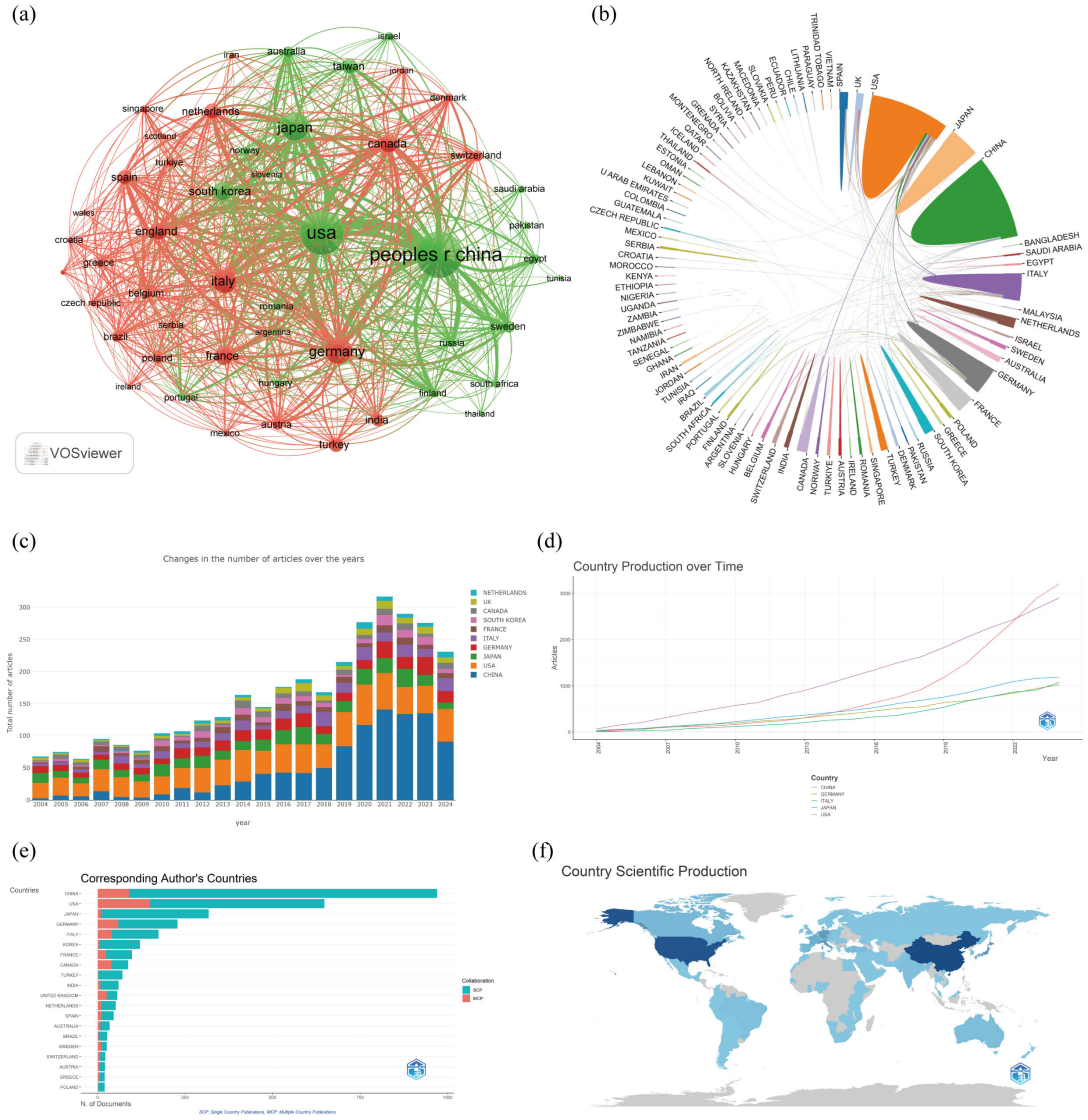
Countries and Institutions analysis. **(a)** A network visualization map between countries; **(b)** Map of contributions to publications by country; **(c)** Bar chart of the annual number of published documents in different countries; **(d)** The publication volume of the top 5 country over time; **(e)** Distribution of corresponding authors’ countries and cooperation; **(f)** Geographical distribution of the countries in terms of publications.

A total of 92 countries contributed to these publications. The top 10 publications were dominated by Asia (n=4), the Americas (n=2), Europe (n=3), and Eurasia (n=1), with Asia standing out as particularly prominent. China had the highest number of publications (n=970, 29%), followed by the United States (n=648, 19.4%), Japan (n=317, 9.5%), and Germany (n=228, 6.8%) (**Figures 3a, b**; **Table 1**). The annual publication output of the United States had seen rapid and steady growth, while China had significantly increased its number of publications annually since 2019 (**Figures 3c, d**). Canada’s international collaboration rate was significantly higher at 44.8% compared to China (9.3%), the United States (23.1%), and Japan (2.8%), suggesting that Canada had a more extensive international collaboration network. The publication with the highest number of contributions from China requires further improvement in its international collaboration efforts (**Figures 3e, f**).

**Table 1:** Top 10 countries on research of liver cancer with bone metastasis from 2004 to 2024.

Rank	Country	Articles	Articles %	SCP	MCP	MCP%
1	CHINA (Asia)	970	29.0	880	90	9.30
2	USA (North America)	648	19.4	498	150	23.1
3	JAPAN (Asia)	317	9.50	308	9	2.80
4	GERMANY (Europe)	228	6.80	169	59	25.9
5	ITALY (Europe)	174	5.20	134	40	23.0
6	KOREA (Asia)	121	3.60	116	5	4.10
7	FRANCE (Europe)	98	2.90	74	24	24.5
8	CANADA (North America)	87	2.60	48	39	44.8
9	TURKEY (Asia Europe)	71	2.10	69	2	2.80
10	INDIA (South Asia)	60	1.80	52	8	13.3

### 3.3 Analysis of the contributions of organizations

**Figure 4:**
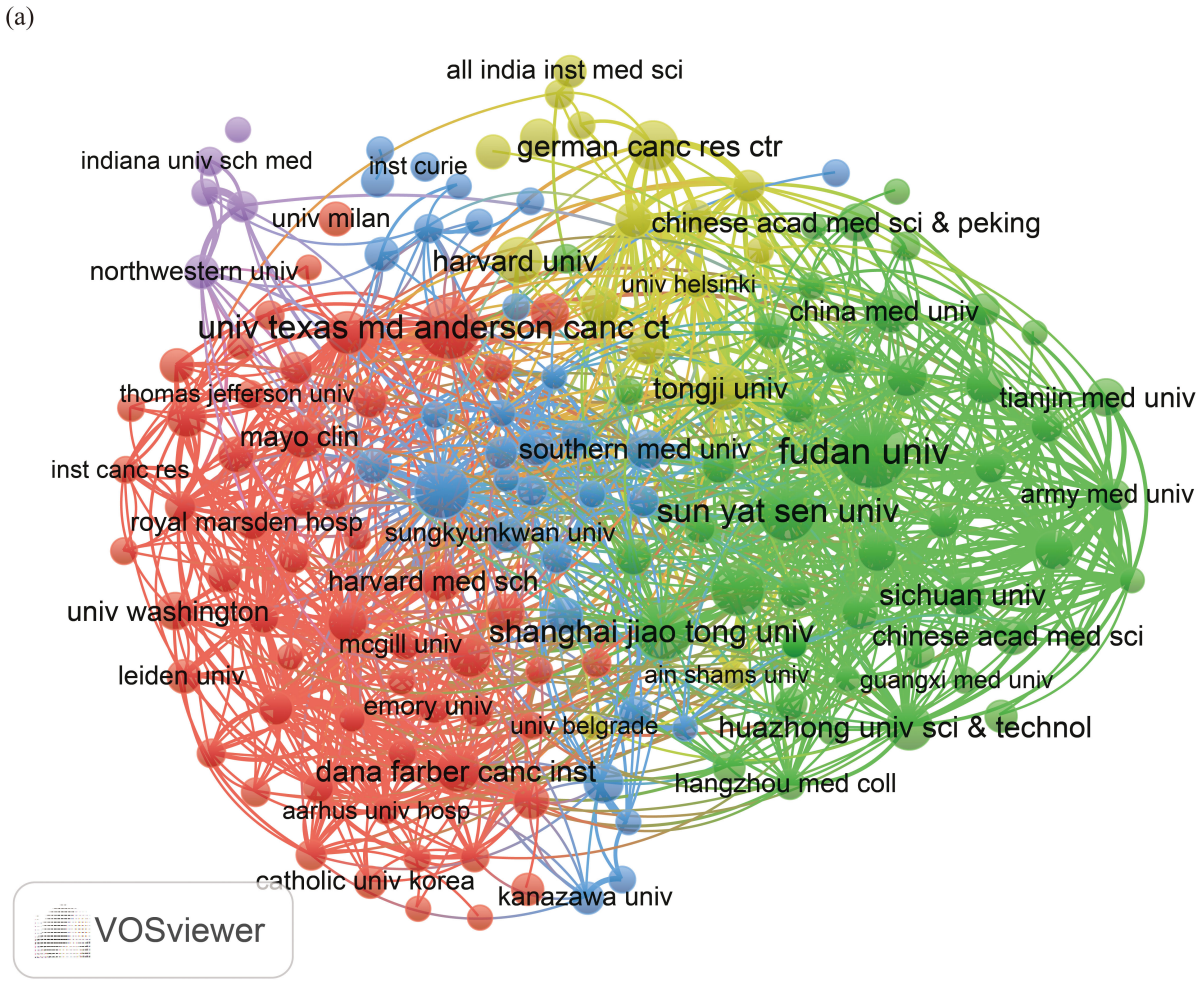
**(a)** A network visualization map between organizations.

We established a minimum requirement of 5 publications and utilized VOSviewer to identify a total of 13,188 organizations. Using the publication counts and the relationships among organizations, we created a visual collaboration network divided into 5 clusters, as illustrated in **Figure 4a**. The top 10 publishing organizations were primarily from 3 countries: China (n = 6), the United States (n = 3), and Germany (n = 1), with publication rates of 10.6%, 5.14%, and 1.39%, respectively (**Table 2**).

**Table 2:** Top 10 organizations on research of liver cancer with bone metastasis from 2004 to 2024.

Rank	Organization	Documents	Documents%	Citations
1	Fudan University (China)	79	2.90	1833
2	University of Texas MD Anderson Cancer Center (USA)	60	2.20	5043
3	Sun Yat-sen University (China)	51	1.87	1430
4	Shanghai Jiao Tong University (China)	49	1.80	748
5	Memorial Sloan Kettering Cancer Center (USA)	46	1.69	9592
6	Zhejiang University (China)	44	1.61	371
7	German Cancer Research Center (German)	38	1.39	1872
8	Harvard University (USA)	34	1.25	2765
9	Tongji University (China)	34	1.25	697
10	Huazhong University of Science and Technology (China)	32	1.17	313

### 3.4 Analysis of the contributions of journals

**Figure 5:**
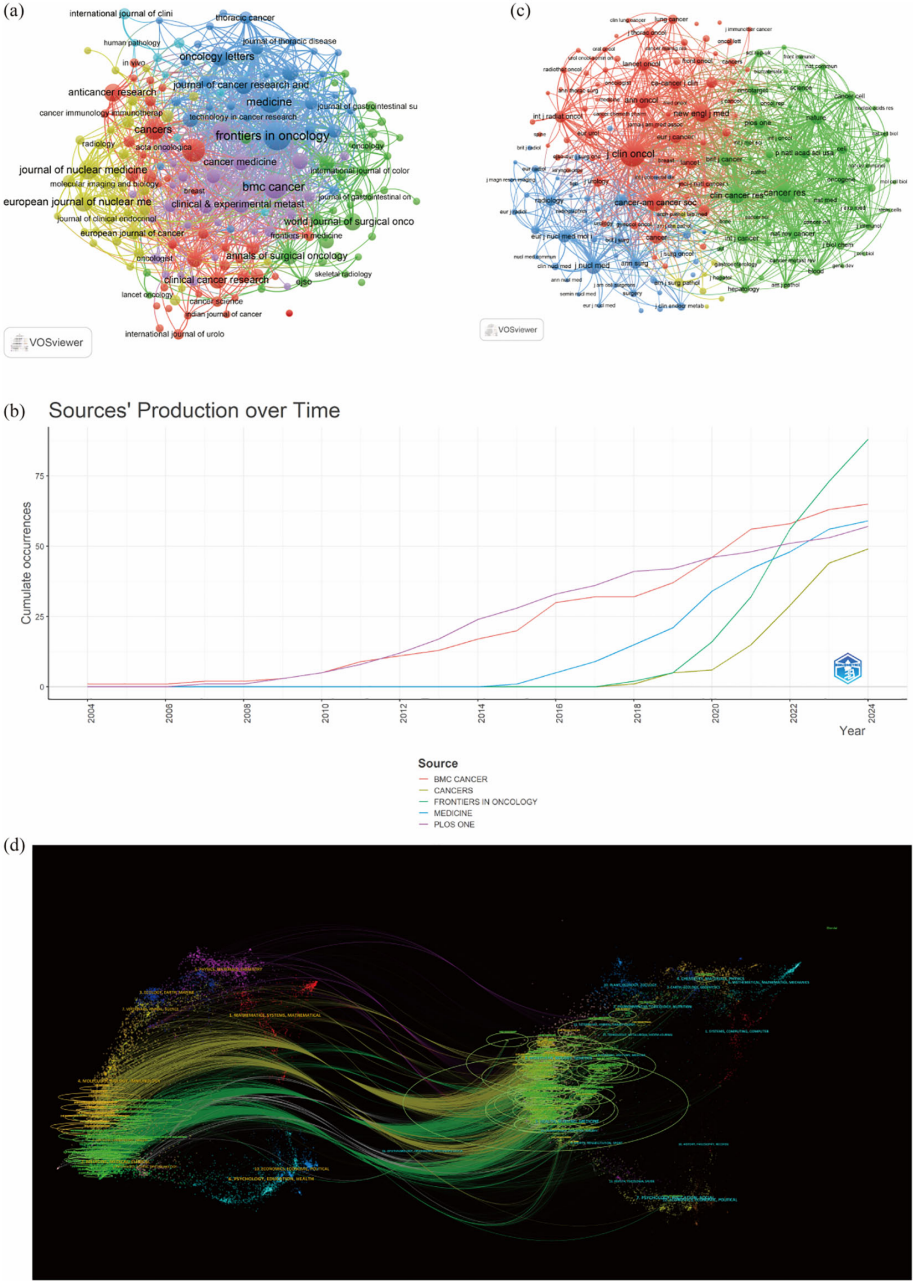
**(a)** Citation-journals network; **(b)** The publication volume of the top 5 journals over time; **(c)** Co-citation journals network: **(d)** The dual-map overlay of journals on research.

A total of 830 journals have published research on liver cancer with bone metastasis. The journal with the highest number of publications was “Frontiers in Oncology” (n=88, 3.86%), followed by “BMC Cancer” (n=65, 2.86%), “Medicine” (n=59, 2.59%), and “PLOS ONE” (n=57, 2.5%). Among the top 10 journals ranked by impact factor, the highest was the Journal of Nuclear Medicine (IF = 9.1), followed by the European Journal of Nuclear Medicine and Molecular Imaging (IF = 8.6) and Frontiers in Oncology (IF = 6.24). Following this, we filtered 187 journals that had at least five related publications and constructed a journal network, additionally, we observed strong cooperation among countries and various institutions (**Table 3**; **Figure 5a**). **Figure 5b** showed the publication curve of the top 5 journals from 2004 to 2024. **Table 4** revealed that all of the top 10 co-cited journals received over 1000 citations. “J Clin Oncol” led with 4275 citations, followed by “Cancer Res” with 2421 citations.

**Table 3:** Journals for research of liver cancer with bone metastasis from 2004 to 2024.

Rank	Journals	Documents	Documents%	Citations	IF
1	frontiers in oncology	88	3.86%	641	6.24
2	BMC cancer	65	2.85%	1766	3.4
3	medicine	59	2.59%	304	1.6
4	PLOS one	57	2.50%	1567	2.9
5	cancers	49	2.15%	589	6.1
6	journal of nuclear medicine	43	1.89%	3174	9.1
7	oncology letters	41	1.80%	494	2.5
8	European journal of nuclear medicine and molecular imaging	40	1.75%	3444	8.6
9	journal of cancer research and clinical oncology	35	1.54%	484	3.9
10	anticancer research	34	1.49%	325	2.43

**Table 4:** Co-cited journals for research of liver cancer with bone metastasis from 2004 to 2024.

Rank	Co-cited journals	Citations	IF
1	j clin oncol	4275	44.54
2	cancer res	2421	12.7
3	new engl j med	1983	158.5
4	cancer-am cancer soc	1936	6.86
5	clin cancer res	1639	13.8
6	ann oncol	1572	32.97
7	j nucl med	1532	9.30
8	nature	1092	50.5
9	brit j cancer	1051	6.4
10	lancet oncol	1045	41.41

The next highest citations were for “Cancer Res” (2421), “New Engl J Med” (1983), “Cancer-Am Cancer Soc” (1936), and “Clin Cancer Res” (1639). “New Engl J Med” had the highest impact factor of 158.5, followed by “J Clin Oncol” with an impact factor of 44.54. After this, we filtered 188 journals that had at least 100 related publications and constructed a journal network, as illustrated in **Figure 5c**. The dual map overlay illustrates the citation relationships between journals and their co-cited counterparts. The left side represents the citing journals, while the right side shows the co-cited journals. As showed in **Figure 5d** shows that these two paths are mainly green and yellow, reflecting a diverse array of cited papers. Such as yellow cluster4 (MOLECULAR, BIOLOGY, IMMUNOLOGY), green cluster9(DENTISTRY, BERMATOLOGY, SURGERY), and cluster2 (MEDICINE, MEDICICAL, CLINICAL) journal articles mainly cited to cluster8 (MOLECULAR BIOLOGY, GENETICS) and cluster5(HEALTH, NURSING, MEDICINE), etc.

### 3.5 Analysis of contributions of prolific authors and co-cited authors

**Figure 6:**
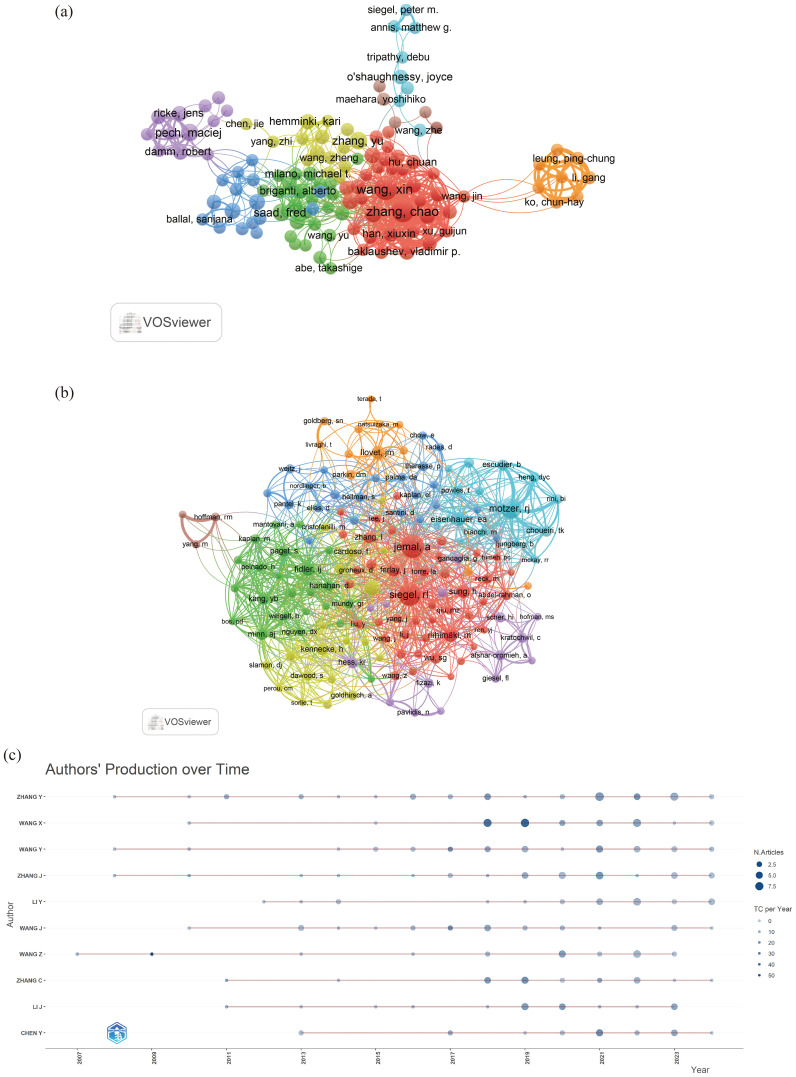
**(a)** A network visualization between authors; **(b)** Co-Cited Authors network; **(c)** A graph of an author’s work over time.

A total of 23,710 authors contributed to research on liver cancer with bone metastasis. Among the top 10 authors by publication count, 6 authors published more than 10 papers. Wang Xin and Zhang Chao had the highest number of publications, each with 18 papers (**Table 5**). We constructed a collaboration network based on authors who had published 5 or more papers (**Figure 6a**). Additionally, close collaboration was noted among several authors. For instance, Wang, Xin and Zhang, Chao had a close collaboration, as did Saad, Fred; Briganti, Alberto; Abdel-Rahman, Omar and Tong, Yuexin. From the 54,118 co-cited authors, we included 153 articles in the visual analysis by setting a minimum citation threshold of 30. There were 9 authors with more than 100 co-citations, with Jemal, A. leading at 313 citations. Active collaboration was also observed among various co-cited authors, including Jemal A., Siegel RL., Motzer RJ., Eisenhauer EA., and Coleman RE. (**Table 6**; **Figure 6b**). **Figure 6c** showed that Wang, Z. was the first to publish on this topic in 2007, followed with Zhang Y.; Wang Y. and Zhang J. who researched the topic in 2008, and they continue to work in this direction to date.

**Table 5:** Authors for research of liver cancer with bone metastasis from 2004 to 2024.

Rank	Authors	Documents	Citations	Total link strength
1	Wang, Xin	18	407	320
2	Zhang, Chao	18	340	321
3	Saad, Fred	10	384	27
4	Zhang, Yu	10	278	7
5	Wang, Guowen	10	254	232
6	Pech, Maciej	10	167	73
7	Debus, Juergen	9	531	15
8	Zhang, Wei	9	506	19
9	Roesch, Frank	9	271	33
10	Koo, Ja Seung	9	225	2

**Table 6:** Co-cited authors for research of liver cancer with bone metastasis from 2004 to 2024.

Rank	Co-cited authors	Citations	Total link strength
1	Jemal, A	313	838
2	Siegel, RL	305	933
3	Motzer, RJ	247	1117
4	Coleman, RE	135	457
5	Riihimäki, M	120	451
6	Eisenhauer, EA	115	395
7	Fidler, IJ	107	561
8	Sung, H	105	297
9	Llovet, JM	103	286
10	Ferlay, J	92	265

### 3.6 Analysis of keywords

**Figure 7:**
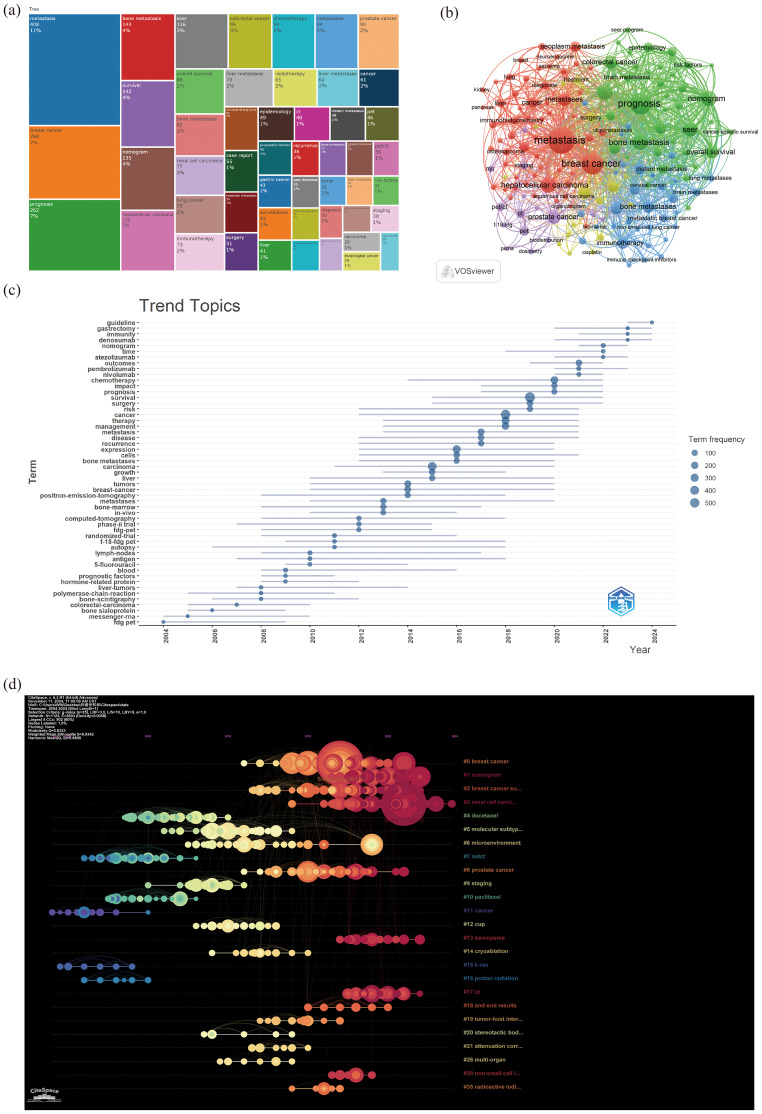
**(a)** Top 50 keywords on research; **(b)** author keywords network; **(c)** keywords trend topics; **(d)** timeline-keywords.

Through the co-occurrence analysis of keywords, we could quickly capture research hotspots in a certain field. **Figure 7a** showed the top 20 high-frequency keywords in research of liver cancer with bone metastasis. Among them, metastasis became the most prominent keyword.

We filtered keywords with the number of occurrences more than or equal to 10 and performed cluster analysis through VOSviewer. The larger the node, the more keyword references; The thicker the line between nodes, the stronger the connection between keywords. As shown in **Figure 7b**, we obtained 8 clusters in total and represented 8 research directions. For example, the keywords in purple clusters consisted of prostate cancer, pet, pet/ct, staging etc. green clusters consisted of prognosis, bone metastasis, nomogram, colorectal cancer, etc. red clusters consisted of metastasis, breast cancer, hepatocellular carcinoma, metastases, etc. blue clusters consisted of bone metastases, immunotherapy, breast cancer, brain metastases, lung metastases, etc.

The trend topic analysis of the keywords (**Table 7**; **Figure 7c**) showed that from 2004 to 2009, the research in this period mainly focused on fdg pet, messenger-rna, bone sialoprotein, polymerase-chain-reaction, liver-tumors, hormone-related protein, prognostic factors, etc. Since 2010, there had been an explosion of research interest in this direction, mainly involving survival, cancer, therapy, management, expression, metastases, bone-marrow, chemotherapy, etc; In addition, from 2022 to 2024, keywords such as “immunity,” “gastrectomy,” and “guidelines” had frequently appeared. And the emerging drug denosumab might represent the current research hotspot of liver cancer with bone metastasis.

**Table 7:** Authors keywords for research of liver cancer with bone metastasis from 2004 to 2024.

Rank	Keywords	Frequency	Year (Q1)	Year (Median)	Year (Q3)
1	metastasis	408	2013	2017	2021
2	breast cancer	268	2012	2016	2021
3	prognosis	262	2018	2020	2022
4	bone metastasis	143	2016	2020	2022
5	survival	142	2017	2020	2022
6	nomogram	135	2021	2022	2023
7	hepatocellular carcinoma	130	2010	2016	2020
8	seer	116	2020	2021	2023
9	colorectal cancer	95	2015	2018	2021
10	metastases	94	2011	2016	2021

A timeline image could display the time periods and trends for different research keywords. The term co-occurrence analysis revealed a total of 35 major clusters, including “#0breast cancer”, “#1nomogram”, “#2breast cancer subtype”, “#3renal cell carcinoma”, “#4docetaxel”, “#5molecular subtype”, and “#6microenvironment”, among others. Moreover, the research interest in #0, #1, #2, and #3 had surged since 2010 (**Figure 7d**).

### 3.7 Co-cited references and citation bursts

**Figure 8:**
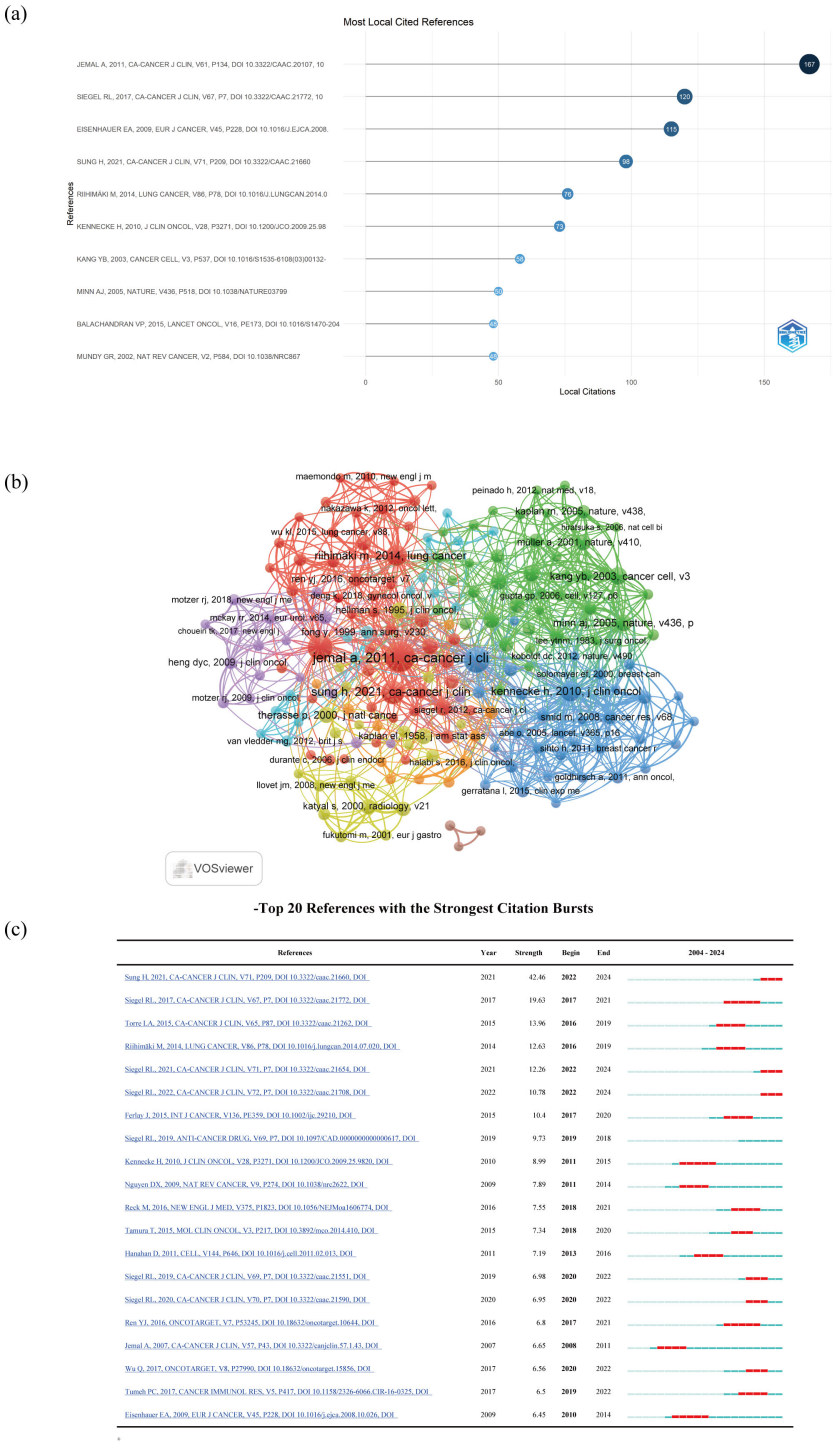
**(a)** Top 10 co-cited references; **(b)** The visualization of cited references; **(c)** Top 20 References with the Strongest Citation Bursts.

The timeline view of clustered maps of 79935 co-cited references from 2004 to 2024. In top 10 co-cited references, there were 3 documents that had been cited more than 100 times, with the highest citation count reaching 167 times (19, 25–33) (**Table 8**; **Figure 8a**). We selected references with co-citation more than or equal to 15 for the construction of the co-citation network map. According to **Figure 8b**, the citation count and total link strength of the paper Jemal A, 2011, CA-Cancer J Clin significantly exceed those of other publications. Reference with citation bursts refers to those references that were frequently cited by scholars in a certain field over a period of time. In our study, 116 references with strong citation bursts were identified by CiteSpace. As shown in **Figure 8c**, every bar represents a year, and the red bar represents strong citation burstiness. Citation bursts for references appeared as early as 2008 and as late as 2022. These 20 references had an overall range of 6.45 to 42.46 for burst strength and 3 to 5 years for endurance strength. The most citation surging paper (intensity=42.46) was “Global Cancer Statistics 2020: GLOBOCAN Estimates of Incidence and Mortality Worldwide for 36 Cancers in 185 Countries (25)” by Sung H et al., published in CA Cancer J Clin in 2021, with an impact factor of 503.1. The citation surge occurred from 2022 to 2024. It described the magnitude and distribution of the overall disease burden and major types of cancer in 2020, briefly comments on the relevant risk factors and the prospects for preventing the major cancers observed globally, and finally predicted the severity of the disease in 2040 based on global population projections.

**Table 8:** Co-cited references for research of liver cancer with bone metastasis from 2004 to 2024.

Rank	Cited reference	Title	Citations
1	jemal a, 2011, ca-cancer j clin, v61, p134 (26)	Global cancer statistics	167
2	siegel rl, 2017, ca-cancer j clin, v67, p7 (27)	Colorectal cancer statistics, 2017	120
3	eisenhauer ea, 2009, eur j cancer, v45, p228 (28)	New response evaluation criteria in solid tumours: revised RECIST guideline (version 1.1)	115
4	sung h, 2021, ca-cancer j clin, v71, p209 (25)	Global Cancer Statistics 2020: GLOBOCAN Estimates of Incidence and Mortality Worldwide for 36 Cancers in 185 Countries	98
5	riihimäki m, 2014, lung cancer, v86, p78 (29)	Metastatic sites and survival in lung cancer	76
6	kennecke h, 2010, j clin oncol, v28, p3271 (30)	Metastatic behavior of breast cancer subtypes	73
7	kang yb, 2003, cancer cell, v3, p537 (31)	A multigenic program mediating breast cancer metastasis to bone	58
8	minn aj, 2005, nature, v436, p518 (32)	Genes that mediate breast cancer metastasis to lung	50
9	balachandran vp, 2015, lancet oncol, v16, pe173 (33)	Nomograms in oncology: more than meets the eye	48
10	mundy gr, 2002, nat rev cancer, v2, p584 (19)	Metastasis to bone: causes, consequences and therapeutic opportunities	48

## 4 Discussion

### 4.1 Global research status and trends

Based on the statistical analysis of this study, China was the country with the highest number of published papers, accounting for 29% of the total literature, which might be related to the highest incidence of liver cancer in China. In terms of international collaboration rate, Canada ranked the highest, far surpassing China, the United States, Japan, and others, indicating that these major contributing countries needed to strengthen international exchange and cooperation. In the top 10 institutional contribution analysis, Chinese universities had made the greatest contribution (n=6), including Fudan University, Sun Yat-sen University, Shanghai Jiao Tong University, Zhejiang University, Tongji University, and Huazhong University of Science and Technology; followed by American institutions (n=3), such as The University of Texas MD Anderson Cancer Center, Memorial Sloan Kettering Cancer Center, and Harvard University. In terms of journal publication volume and citation frequency, the top 10 journals had a total co-citation count of over 1000, with “Frontiers In Oncology” having the highest output; “European Journal of Nuclear Medicine and Molecular Imaging” had the highest citation rate, and the journal with the highest impact factor was “New Engl J Med” (IF=158.5), demonstrating the importance and reference value of the papers. The dual-map coverage indicated that these journals cover a wide range of fields, including molecular, biological, immunological, cosmetic, surgical, pharmaceutical, medical, nursing, and clinical disciplines. A total of 23,710 authors had participated in research related to liver cancer with bone metastasis, with Wang, Xin and Zhang, Chao being the individual authors who had published the most papers. There had been 54,118 co-cited authors, with Jemal, A (n=313) having the highest number of citations, indicating active collaborative relationships among different authors. Wang, Z was the first to publish research on this topic in 2007, while Zhang Y, Wang Y, and Zhang J conducted research on this topic simultaneously in 2008 and had been actively involved in this research direction to date.

### 4.2 Hotspots and development trends

Current diagnostic research in liver cancer bone metastasis primarily centers on molecular markers and imaging technologies. Keywords such as “PET/CT,” “nomogram,” and “serum lnc34a” (**Table 7**) highlight the emphasis on non-invasive detection and prognostic modeling. Studies have explored functional imaging for metastatic site identification and serum biomarkers (e.g., lncRNAs) for early metastasis prediction. However, diagnostic gaps persist: (1) Limited sensitivity of current imaging in detecting micro-metastases; (2) Lack of standardized molecular markers for routine clinical use; (3) Inconsistent diagnostic criteria across studies, hindering cross-validity. The emerging trend toward multi-omics integration (e.g., genomics and radiomics) aims to address these gaps but remains in the preclinical stage.

We had identified and summarized the research hotspots and frontiers of bone metastasis from liver cancer through the analysis of cited literature, citation bursts, keyword clustering, and keyword topic trends in liver cancer with bone metastasis. Our study highlighted the necessity of liver cancer treatment methods as well as the early monitoring and identification of bone metastasis, involving the following aspects:

Firstly, through keyword co-occurrence analysis, the research had been clustered into eight directions, with “metastasis” being the most prominent keyword. This included cancer detection and treatment methods such as PET, PET/CT, radiotherapy, immunotherapy, targeted therapy, and drugs; types of cancer metastasis such as breast cancer, hepatocellular carcinoma, and colorectal cancer; sites of cancer metastasis involving bone, brain, and lung metastases. Meanwhile, the timeline visualization could display the time periods and trends of different research keywords, revealing a total of 35 main clusters. Since 2010, there had been a significant increase in research interest related to #0 breast cancer, #1 nomogram, #2 breast cancer subtypes, and #3 renal cell carcinoma. The emerging drug denosumab may be a current research hot spot for the treatment of liver cancer with bone metastasis (21, 34–39).

Secondly, the literature construction network graph showed that the paper titled “Global Cancer Statistics (26) “ by Jemal A, et al., published in the CA-Cancer J Clin journal in 2011, had the highest number of citations (n=167) and the highest impact factor (IF 503.1), with a Web of Science (WOS) quartile of Q1 and a total link strength of 364. This paper analyzed cancer cases for the year based on GLOBOCAN 2008 and compared them with common causes of cancer death over the past decade, suggested that the low survival rate of cancer was likely related to late diagnosis and limited opportunities for timely or standard treatment. It highlighted the positive role of promoting cancer control knowledge, such as implementing tobacco control, vaccination (for liver cancer and cervical cancer), early detection and treatment plans, as well as public health campaigns promoting physical activity and healthier dietary intake in preventing the global cancer burden.

Thirdly, CiteSpace identified 116 papers with strong citation bursts, with the earliest citation burst appeared in 2008 and the latest in 2022. The overall range of burst strength for the top 20 references was from 6.45 to 42.46, and the overall range of endurance strength was from 3 to 5 years. The paper with the highest citation strength (strength=42.46) was “Global Cancer Statistics 2020: GLOBOCAN Estimates of Incidence and Mortality of 36 Cancers in 185 Countries Worldwide (25)” by Sung H et al., published in CA Cancer J Clin in 2021, with an impact factor of 503.1 and the citation burst occurred from 2022 to 2024. It conducted a statistical analysis of the size and distribution of the overall global cancer burden and types of cancer, and predicted the severity of the disease in 2040, guiding research in the field.

### 4.3 Strengths and limitations

This study was the first to employ bibliometric methods to analyze the current research status of liver cancer with bone metastasis and use three bibliometric tools for data visualization, which helped researchers better understand the field and explored new research directions. However, there were some limitations. First, we only selected the Web of Science (WOS) database and English languages as the data source, which might result in the omission of other research. Second, there were significant differences in the incidence rate of liver cancer, diagnostic methods and treatment drugs among countries, and accordingly, research focuses and advancements were also varied. Finally, recent high-quality articles might not receive sufficient attention due to their short publication time and low citation frequency. For example, the International Agency for Research on Cancer (IARC) published the latest global cancer statistics for 2022 in an article in May 2024 (“Global Cancer Statistics 2022: GLOBOCAN Estimates of Incidence and Mortality for 36 Cancers in 185 Countries Worldwide”) and this piece was overlooked precisely because of this reason, despite its high quality (40). however, due to the varying cancer incidence rates in each country, indicated the necessity to combine with the specific circumstances of each country. In summary, despite these limitations, our study offered a comprehensive overview of the clinical research, monitoring/treatment methods and prognosis of liver cancer with bone metastasis, which were also of significant value for understanding the current research trends in this field.

## 5 Conclusions

Our bibliometric study of liver cancer with bone metastasis shows that this field is gaining increasing attention from researchers. We identified key areas for future research, including pathogenesis, detection technologies, radiotherapy, targeted therapy, immunotherapy, bone protective agents, and prognostic analysis. The integration of individualized treatment plans has significantly improved clinical efficacy for patients. Expanding surgical indications and standardizing clinical management techniques have increased patient survival times, while conversion therapy has provided critically ill patients with greater surgical opportunities. We must emphasize the importance of early detection and comprehensive disease management to prevent progression and improve prognosis. Future research will aim to enhance early screening accuracy, optimize treatment plans, and investigate new therapeutic targets.

## References

[1] Bray F Laversanne M Sung H Ferlay J Siegel RL Soerjomataram I. Global cancer statistics 2022: GLOBOCAN estimates of incidence and mortality worldwide for 36 cancers in 185 countries. CA: Cancer J Clin. (2024) 74:229–63. doi: 10.3322/caac.21834, PMID: 38572751

[2] Feng M Pan Y Kong R Shu S. Therapy of primary liver cancer. Innovation (Cambridge (Mass)). (2020) 1:100032. doi: 10.1016/j.xinn.2020.100032, PMID: 32914142 PMC7416132

[3] Are C Meyer B Stack A Ahmad H Smith L Qian B. Global trends in the burden of liver cancer. J Surg Oncol. (2017) 115:591–602. doi: 10.1002/jso.24518, PMID: 28345140

[4] Jin X Ma X Zhao D Yang L Ma N. Immune microenvironment and therapeutic progress of recurrent hepatocellular carcinoma after liver transplantation. Trans Oncol. (2023) 28:101603. doi: 10.1016/j.tranon.2022.101603, PMID: 36542991 PMC9794975

[5] Anwanwan D Singh SK Singh S Saikam V Singh R. Challenges in liver cancer and possible treatment approaches. Biochim Biophys Acta Rev Cancer. (2020) 1873:188314. doi: 10.1016/j.bbcan.2019.188314, PMID: 31682895 PMC6981221

[6] Lee HY Hong IS. Targeting liver cancer stem cells: an alternative therapeutic approach for liver cancer. Cancers. (2020) 12(10):2746. doi: 10.3390/cancers12102746, PMID: 32987767 PMC7598600

[7] Habito CM. Editorial: radioembolization in the treatment of liver cancer: A multidisciplinary approach for individualized therapy. Front Oncol. (2015) 5:216. doi: 10.3389/fonc.2015.00216, PMID: 26500889 PMC4597107

[8] López-Pelayo H Altamirano J López E Barrio P López A Gual A. Role of Alcohol and Drug Detection by Regular Urine Sample Testing in pre-transplant evaluation for Alcohol Liver Disease. Adicciones. (2020) 32:243–54. doi: 10.20882/adicciones.1121, PMID: 30059586

[9] Wang M Peng P Chen Z Deng X. Nanoparticle delivery of active traditional Chinese medicine ingredients: A new strategy for the treatment of liver cancer. Curr Pharm Biotechnol. (2023) 24:1630–44. doi: 10.2174/1389201024666230313151316, PMID: 36915990

[10] Peng Y Wu X Zhang Y Yin Y Chen X Zheng D. An overview of traditional Chinese medicine in the treatment after radical resection of hepatocellular carcinoma. J Hepatocell Carcinoma. (2023) 10:2305–21. doi: 10.2147/JHC.S413996, PMID: 38143910 PMC10743783

[11] Wang D Xiao M Wan ZM Lin X Li QY Zheng SS. Surgical treatment for recurrent hepatocellular carcinoma: Current status and challenges. World J Gastrointest Surg. (2023) 15:544–52. doi: 10.4240/wjgs.v15.i4.544, PMID: 37206072 PMC10190723

[12] Joliat GR Labgaa I Uldry E Demartines N Halkic N. Recurrence rate and overall survival of operated ruptured hepatocellular carcinomas. Eur J Gastroenterol Hepatol. (2018) 30:792–6. doi: 10.1097/MEG.0000000000001115, PMID: 29538038

[13] Takei D Tagami K. Management of cancer pain due to bone metastasis. J Bone Mineral Metab. (2023) 41:327–36. doi: 10.1007/s00774-022-01382-y, PMID: 36418587

[14] Riffel RM Göbel A Rachner TD. Bone metastases: from mechanisms to treatment. Semin Oncol Nurs. (2022) 38:151277. doi: 10.1016/j.soncn.2022.151277, PMID: 35474145

[15] Ban J Fock V Aryee DNT Kovar H. Mechanisms, diagnosis and treatment of bone metastases. Cells. (2021) 10(11):2944. doi: 10.3390/cells10112944, PMID: 34831167 PMC8616226

[16] Wang M Xia F Wei Y Wei X. Molecular mechanisms and clinical management of cancer bone metastasis. Bone Res. (2020) 8:30. doi: 10.1038/s41413-020-00105-1, PMID: 32793401 PMC7391760

[17] Jing D Zhao Q Zhao Y Lu X Feng Y Zhao B. Management of pain in patients with bone metastases. Front Oncol. (2023) 13:1156618. doi: 10.3389/fonc.2023.1156618, PMID: 37007073 PMC10063159

[18] Zhang L Niu H Yang P Ma J Yuan BY Zeng ZC. Serum lnc34a is a potential prediction biomarker for bone metastasis in hepatocellular carcinoma patients. BMC Cancer. (2021) 21:161. doi: 10.1186/s12885-021-07808-6, PMID: 33588789 PMC7885499

[19] Mundy GR. Metastasis to bone: causes, consequences and therapeutic opportunities. Nat Rev Cancer. (2002) 2:584–93. doi: 10.1038/nrc867, PMID: 12154351

[20] Bonetto R Tallet A Mélot A Calderon B Barlesi F. The management of bone metastasis]. Bull du Cancer. (2017) 104:585–92. doi: 10.1016/j.bulcan.2017.02.004, PMID: 28391985

[21] Dell’aquila E Armento G Iuliani M Simonetti S D'Onofrio L Zeppola T. Denosumab for cancer-related bone loss. Expert Opin Biol Ther. (2020) 20:1261–74. doi: 10.1080/14712598.2020.1814731, PMID: 32835531

[22] Van Eck NJ Waltman L. Software survey: VOSviewer, a computer program for bibliometric mapping. Scientometrics. (2010) 84:523–38. doi: 10.1007/s11192-009-0146-3, PMID: 20585380 PMC2883932

[23] Synnestvedt MB Chen C Holmes JH. CiteSpace II: visualization and knowledge discovery in bibliographic databases. AMIA Annu Symposium Proc AMIA Symposium. (2005) 2005:724–8., PMID: 16779135 PMC1560567

[24] Aria M Cuccurullo C. bibliometrix: An R-tool for comprehensive science mapping analysis. J Informetrics. (2017) 11:959–75. doi: 10.1016/j.joi.2017.08.007

[25] Sung H Ferlay J Siegel RL Laversanne M Soerjomataram I Jemal A. Global cancer statistics 2020: GLOBOCAN estimates of incidence and mortality worldwide for 36 cancers in 185 countries. CA: Cancer J Clin. (2021) 71:209–49. doi: 10.3322/caac.21660, PMID: 33538338

[26] Jemal A Bray F Center MM Ferlay J Ward E Forman D. Global cancer statistics. CA: Cancer J Clin. (2011) 61:69–90. doi: 10.3322/caac.20107, PMID: 21296855

[27] Siegel RL Miller KD Fedewa SA Ahnen DJ Meester RGS Barzi A. Colorectal cancer statistics, 2017. CA: Cancer J Clin. (2017) 67:177–93. doi: 10.3322/caac.21395, PMID: 28248415

[28] Eisenhauer EA Therasse P Bogaerts J Schwartz LH Sargent D Ford R. New response evaluation criteria in solid tumours: revised RECIST guideline (version 1.1). Eur J Cancer (Oxford England: 1990). (2009) 45:228–47. doi: 10.1016/j.ejca.2008.10.026, PMID: 19097774

[29] Riihimäki M Hemminki A Fallah M Thomsen H Sundquist K Sundquist J. Metastatic sites and survival in lung cancer. Lung Cancer (Amsterdam Netherlands). (2014) 86:78–84. doi: 10.1016/j.lungcan.2014.07.020, PMID: 25130083

[30] Kennecke H Yerushalmi R Woods R Cheang MC Voduc D Speers CH. Metastatic behavior of breast cancer subtypes. J Clin Oncol: Off J Am Soc Clin Oncol. (2010) 28:3271–7. doi: 10.1200/JCO.2009.25.9820, PMID: 20498394

[31] Kang Y Siegel PM Shu W Drobnjak M Kakonen SM Cordón-Cardo C. A multigenic program mediating breast cancer metastasis to bone. Cancer Cell. (2003) 3:537–49. doi: 10.1016/S1535-6108(03)00132-6, PMID: 12842083

[32] Minn AJ Gupta GP Siegel PM Bos PD Shu W Giri DD. Genes that mediate breast cancer metastasis to lung. Nature. (2005) 436:518–24. doi: 10.1038/nature03799, PMID: 16049480 PMC1283098

[33] Balachandran VP Gonen M Smith JJ DeMatteo RP. Nomograms in oncology: more than meets the eye. Lancet Oncol. (2015) 16:e173–80. doi: 10.1016/S1470-2045(14)71116-7, PMID: 25846097 PMC4465353

[34] Lujambio A Maina F. Turning up our understanding of liver cancer by a notch. J Hepatol. (2021) 74:502–4. doi: 10.1016/j.jhep.2020.10.027, PMID: 33342548

[35] Nakatsukasa K Koyama H Ouchi Y Sakaguchi K Fujita Y Matsuda T. Predictive factors for the efficacy of denosumab in postmenopausal Japanese women with non-metastatic breast cancer receiving adjuvant aromatase inhibitors: a combined analysis of two prospective clinical trials. J Bone Mineral Metab. (2019) 37:864–70. doi: 10.1007/s00774-018-00985-8, PMID: 30868270

[36] Pageau SC. Denosumab. mAbs. (2009) 1:210–5. doi: 10.4161/mabs.1.3.8592, PMID: 20065634 PMC2726593

[37] Deligiorgi MV Trafalis DT. The safety profile of denosumab in oncology beyond the safety of denosumab as an anti-osteoporotic agent: still more to learn. Expert Opin Drug Saf. (2021) 20:191–213. doi: 10.1080/14740338.2021.1861246, PMID: 33287586

[38] Diab DL Watts NB. The use of denosumab in osteoporosis - an update on efficacy and drug safety. Expert Opin Drug Saf. (2024) 23:1069–77. doi: 10.1080/14740338.2024.2386365, PMID: 39262109

[39] Pang KL Low NY Chin KY. A review on the role of denosumab in fracture prevention. Drug Design Dev Ther. (2020) 14:4029–51. doi: 10.2147/DDDT.S270829, PMID: 33061307 PMC7534845

[40] Bray F Laversanne M Sung H Ferlay J Siegel RL Soerjomataram I. Global cancer statistics 2022: GLOBOCAN estimates of incidence and mortality worldwide for 36 cancers in 185 countries. CA Cancer J Clin. (2024) 74:229–63. doi: 10.3322/caac.21834, PMID: 38572751

